# Effects of family genetic risk scores and environmental factors on risk of schizophrenia and bipolar disorder

**DOI:** 10.1038/s41380-026-03715-z

**Published:** 2026-07-03

**Authors:** Natassia Robinson, Alexander Ploner, Henrik Ohlsson, Paul Lichtenstein, Kenneth S. Kendler, Sarah E. Bergen

**Affiliations:** 1 Department of Medical Epidemiology and Biostatistics, Karolinska Institutet, Stockholm, Sweden.; 2 Center for Primary Health Care Research, Lund University, Malmö, Sweden.; 3 Virginia Institute for Psychiatric and Behavioral Genetics, Department of Psychiatry, Virginia Commonwealth University, Richmond, VA, USA.

## Abstract

Both genetic and environmental risk factors contribute to the development of schizophrenia (SCZ) and bipolar disorder (BD), but simultaneous investigation of how these factors influence risk has not yet been comprehensively examined in a large population. Therefore, we aimed to investigate and quantify how a multi-generational index of aggregated genetic risk (family genetic risk scores, FGRS) and environmental exposures jointly contribute to risk for SCZ and BD, and whether these relationships differ between the disorders. We conducted a Swedish register-based matched nested case-control study with 3057 SCZ and 15,029 BD cases diagnosed 1988–2013. We used conditional logistic regression to determine individual and joint effects of established environmental risk factors including adverse childhood experiences (ACEs), substance use, adverse perinatal factors, childhood infections, urban birth and longest residence, and FGRS (quintiles) for SCZ and BD and risk of SCZ/BD. We also estimated population attributable fractions (PAFs) for environmental exposures. FGRS were associated with incremental increases in SCZ and BD, with highest risk observed for the highest quintile (SCZ IRR 9.63, 95% CI 7.17–12.94; BD IRR 6.30, 95% CI 5.80–6.84). FGRS and most environmental exposures were independently associated with risk of SCZ and BD. The greatest PAFs were observed for substance use (SCZ 18.3%; BD 13.2%) and ACEs (SCZ 14.1%; BD 19.8%). FGRS are associated with increased risk for SCZ and BD and were largely independent from environmental risk factors. Potentially modifiable factors, ACEs and substance use, accounted for a moderate proportion of all cases and were particularly impactful for BD (PAF 19.8, 95% CI 18.4, 21.2) and SCZ (PAF 18.3, 95% CI 17.6–19.0), respectively.

## INTRODUCTION

The development of schizophrenia (SCZ) and bipolar disorder (BD) is influenced by combination of genetic and environmental factors, but the interplay between these elements is poorly understood [1]. Family, twin, and molecular genetic studies consistently indicate a high heritability for SCZ and BD and strong genetic correlations between them with numerous shared genetic loci identified [2–4]. Moreover, individuals with a family history of SCZ or BD, particularly first-degree relatives, possess the highest risk for developing the corresponding disorder [2]. However, many studies which integrate family history are limited by their use of a simple binary measure indicating the presence/absence of an affected family member, which is often self-reported and subject to biases. Recently, more comprehensive approaches to capturing genetic risk are being utilized in psychiatric research [5, 6]. These family genetic risk scores (FGRS) are an aggregate measure of genetic risk based on rates of disorder in their immediate and extended relatives, weighted by genetic relatedness (proportion of shared DNA), age at relatives’ diagnosis, cohabitation effects (to account for shared environment), and family size. When family pedigrees are used in combination with national registry diagnostic data, FGRS can offer a high-quality measure of family genetic risk over several generations, including a wider array of relatives, and can be calculated for the entire study population [5]. These scores have provided important insights into genetic architecture of major psychiatric disorders [5] and demonstrate congruence with SCZ and BD diagnostic categories [6]. However, the magnitude of the association between FGRS and risk of SCZ or BD diagnosis has not yet been quantified.

Recently, we investigated a range of environmental factors and risk of SCZ and BD using data from Swedish national registers. We identified several common risk factors including childhood adversity, adverse perinatal factors, substance use, infections, and urban environment (increased risk for SCZ; decreased for BD) [7, 8]. Examination of how a comprehensive measure of familial risk, such as FGRS, impacts the risk associated with these environmental factors remains unexplored in a large population sample for SCZ and BD [1].

While there is a great deal of overlap between the environmental risk factors for SCZ and BD, their interplay with genetic risk may vary, offering a potential means of differentiating between the disorders. However, the extent to which these factors contribute to the overall disorder burden, particularly in relation to familial genetic risks, is unclear and has not been previously attempted at this scale or in relation to FGRS. Identifying the most impactful modifiable environmental exposures through precise measures of absolute risk reduction is necessary to develop effective prevention strategies.

Primary prevention of these disorders requires robust etiological understanding of the genetic and environmental risk factors, and evaluation of their relative contributions to disease risk. Therefore, our objective was to investigate how family genetic risk and environmental exposures jointly contribute to risk for SCZ and BD, and whether these relationships differ between the disorders, by addressing three aims: 1) estimate the risk associated with FGRS, and if this differs between SCZ and BD; 2) examine the joint effects of FGRS and environmental risk factors on risk of SCZ and BD through both additive main effect models and interaction models, as appropriate 3) quantify the population attributable fractions for each of these scenarios to identify potential targets for prevention.

## METHODS

### Study design and data sources

We conducted a register-based, nested case-control study among all persons born in Sweden between 1973 and 1998 (*n* = 2,740,446). This study design embeds a case-control study within a larger cohort followed over time by matching incident cases at their time of diagnosis (index date) with so-far unaffected members of the cohort on potential confounding factors; compared to a full cohort design, nested-case control designs provide superior control of confounding bias while (depending on the number of matched controls) largely preserving power and still allowing estimation of incidence rate ratios.

We linked data from several Swedish population-based registers via unique (anonymized) identification numbers: Total Population Register (TPR), containing demographic data on all individuals resident in Sweden [9]; Multi-Generation Register (MGR), linking individuals born after 1932 to their relatives; National Patient Register (NPR) containing inpatient care in Sweden since 1964 (psychiatric diagnoses from 1973), and outpatient specialty visits since 2001 [10]; and the cause of death register. Additional registers were used to define our exposure variables (supplementary methods). This study was approved by the Regional Ethics Review Board in Stockholm, Sweden (DNR 2013/862–31/5). All methods were performed in accordance with the relevant guidelines and regulations, and no informed consent was required for the analysis of anonymized register data.

To derive our matched cohort, cases of SCZ and BD were identified at their first recorded diagnosis in the NPR at ≥ 15 years old. We did not include individuals with a history of emigration from Sweden in the Migration Register [9] at time of sampling; nor adopted individuals. Each proband case was matched on birth year, sex, and birthplace (county) to five controls without a SCZ or BD diagnosis at the date of diagnosis of their matched case (i.e., incidence density sampling) in line with our prior analyses [7, 8]. Additionally, in this study we applied the extra criterion that individuals were of Swedish ancestry (i.e. with two Swedish-born parents) in order to obtain pedigree information. After exclusions, cases had on average (mean) 4.2 matched controls (Table 1).

### Measures

For both probands and affected relatives, we defined cases using inpatient and outpatient diagnoses in the NPR using ICD-8,9, and 10 codes for SCZ and BD (supplementary methods). Diagnoses were applied without a hierarchy; thus, probands could appear in either matched set.

Environmental exposures are defined in Table 2 and were investigated as binary measures of exposed/unexposed (Table S1). Adjustment for socioeconomic status included highest parental education and household disposable income at age 14–16 (Table S2).

Using the MGR, first through fourth degree relatives of study participants were identified (mean 22 relatives per proband) (Table 1). FGRS for SCZ and BD were calculated using the same seven-step algorithm previously described in Kendler, et al. [5], Supplementary Table 2, including calculations for weights and constants, as originally described by one of the authors (HO). Briefly, the binary disorder trait is transformed into an underlying liability distribution, and mean liability scores are calculated for all relatives, with and without the trait. Weights are calculated which account for the relatives' ages, their genetic relatedness to the proband, and cohabitation effects (shared environment for first-degree relatives). The raw FGRS for each proband is then calculated as the mean of the weighted liability scores across all the proband’s relatives, with a perproband shrinkage factor applied to account for family size. These raw FGRS values are then standardised by proband birth year. We transformed positive FGRS values (probands with at least one affected relative) to quintiles of risk (Figure S1), with probands with negative FGRS (no affected relatives) serving as the reference group.

### Statistical analysis

Descriptive statistics for each exposure are presented as counts and percentages by FGRS quintile and case-control status (Table S3–4). Pearson correlations between all exposures and FGRS were investigated in the SCZ and BD populations. All exposures had low (r < 0.1) correlations with FGRS (Figure S2). Conditional logistic regression was used to calculate all odds ratios, interpreted as incidence rate ratios (IRR), and 95% confidence intervals (CI) for risk of SCZ and BD. *P*-values ≤ 0.05 were considered statistically significant.

First, we estimated the association between FGRS (quintiles) on risk of SCZ and BD relative to those with no affected relatives, with two-sided Wald-test to evaluate the differences between the SCZ and BD risk curves. To explore joint effects, we examined models for each individual exposure unadjusted and adjusted for FGRS. In addition to calculating the relative risk, we calculated the population attributable fraction (PAF) to quantify the theoretical reduction in cases if the population was entirely unexposed to a risk factor [11]. We compared the PAFs for each exposure across each of the statistical models, using the Dahlqwist, et al., method which calculates confounder adjusted PAF estimates for a case-control study taking into account the matched design [12].

We performed three sensitivity analyses to assess the robustness of our findings: 1) substituting FGRS with parental SCZ/BD; 2) exploring multiplicative interactions; and 3) adjustment for childhood socioeconomic status (SES). Given that parental psychopathology may have a more direct influence on environmental exposures, we examined the potential confounding effect of parental SCZ/BD instead of SCZ/BD FGRS. We estimated models with an exposure × FGRS interaction term; these are not intended as a formal interaction analysis, but to assess the robustness of our findings: we allow for FGRS quintile-specific effects of environmental exposures only to quantify how strongly these deviate from the result of our adjusted (main-effect only) models. Results of interaction models are presented as IRRs compared to individuals with no affected relatives and no environmental exposure (roughly approximating the general population). We compared main effect models and interaction models in terms of IRRs, PAFs, and via likelihood ratio tests (LRT). Finally, to obtain more reliable estimates of the absolute risk reduction, we calculated PAFs adjusted for SES as a potential confounder. We present adjustment for SES as a sensitivity analysis to assess the robustness of the results under different assumptions, since in the context of these analyses parental SES could be a mediator (parental psychopathy → lower parental SES → proband psychopathy), or a confounder (direct effect parental SCZ/BD on proband SCZ/BD, not mediated via SES). In prior studies, the associations between these exposures and risk of SCZ/BD were robust to adjustment by SES [7, 8].

## RESULTS

We identified 3057 SCZ cases (matched to 17,532 controls) and 15,029 BD cases (78,640 matched controls) during our study period (Table S2). Most SCZ cases were male (66%), and most BD cases female (66%). Median age at first registered diagnosis was 25.1 for SCZ (IQR 21.4–29.2) and 25.2 for BD (IQR 20.9–30.4).

We observed some differences in the prevalence of exposure to environmental factors across FGRS quintiles (Table S3–4). Individuals with a family history of SCZ and BD, or affected parents, exhibited higher rates of ACEs, with higher prevalence in cases than controls. SCZ and BD cases had high rates of substance use regardless of family history. Higher FGRS correlated with increased urban exposure in BD cases and controls, and to a lesser extent in SCZ cases.

### FGRS and risk of SCZ/BD

**Fig. 1:**
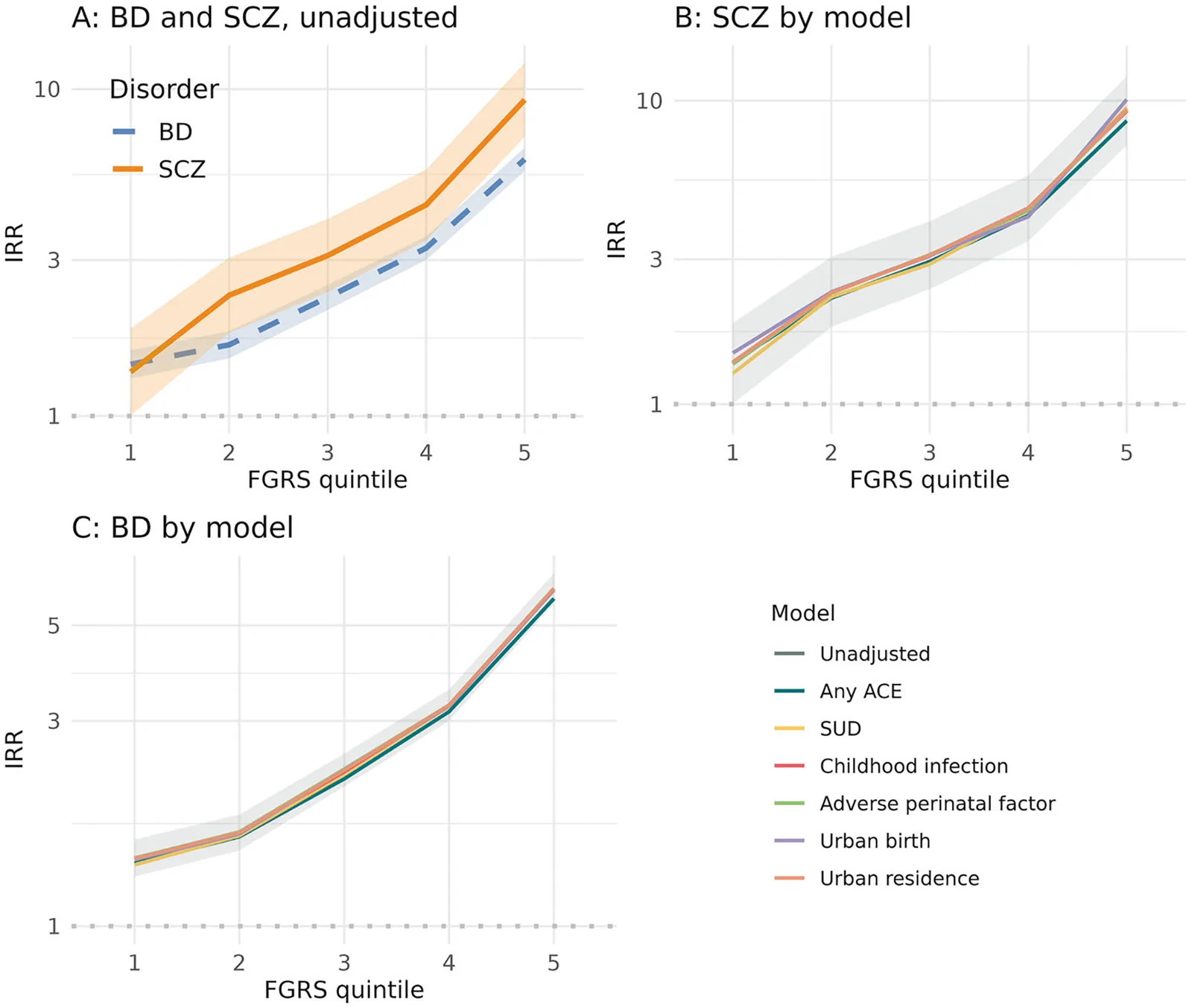
FGRS quintiles and risk for SCZ and BD, unadjusted and adjusted for each risk factor. **A** Association between FGRS and risk (IRR) of SCZ (solid line) and BD (dashed line). Estimates for FGRS quintiles are relative to no affected family members with SCZ or BD. Solid lines represent IRR, and shaded areas show the corresponding 95% confidence intervals. Wald-test to assess differences in the SCZ and BD curves *p* < 0.001. Y-axis is on the log scale. Association between FGRS and risk (IRR) of SCZ (**B**) and BD (**C**) adjusted for each environmental risk factor. Estimates for FGRS quintiles are relative to no affected family members with SCZ or BD. solid lines represent IRR. Shaded areas show the 95% confidence intervals for the unadjusted model. Y-axis is on the log scale.

For both SCZ and BD, we found incremental increases in respective disease risk for exposed individuals with increasing FGRS quintile (Fig. 1A) compared to unexposed individuals with no affected relatives. SCZ FGRS was associated with higher risk of SCZ than BD FGRS was with BD risk, particularly for those in the highest FGRS quintile (Q5 SCZ IRR 9.63; Q5 BD IRR 6.30, Wald-test *p* = 0.003) (Table S5–6).

### Estimating joint effects

In the adjusted model, for both SCZ and BD, controlling for the effect of FGRS did not lead to large changes in estimated disorder risk (Table 3, Figure S3); the largest change was seen for ACEs, where the estimates for both disorders were slightly attenuated (6–7% decrease). Conversely, all of the (exposure) adjusted estimates showed minimal deviation from the unadjusted model, falling within its 95% confidence intervals (Fig. 1B, C).

### Population attributable fractions

**Fig. 2:**
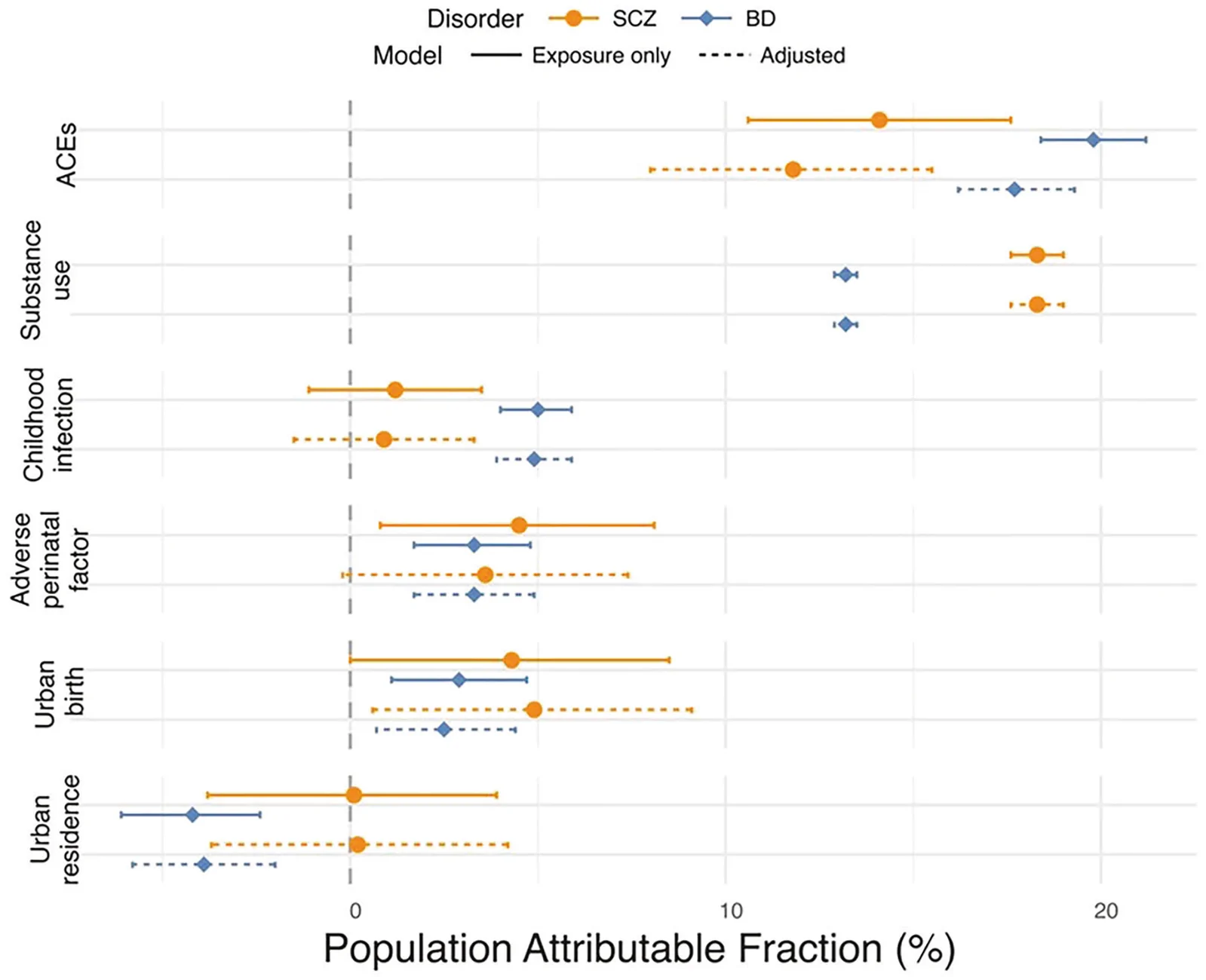
PAFs for environmental risk factors for the crude and FGRS-adjusted models for outcomes SCZ and BD. Points represent IRR, and bars show the corresponding 95% confidence intervals.

We calculated the PAFs for the risk factors for each of the examined models, unadjusted and adjusted for FGRS, to assess how exposures contribute to disorder burden (Fig. 2, Table S7). Both ACEs and substance use had large PAFs for both disorders. Theoretically, eradicating substance use could alleviate the greatest proportion of SCZ cases (18.3%, 7.6–19.0), while eliminating ACEs could remove the largest proportion of BD cases (19.8%, 18.4–21.2). PAFs for adverse perinatal factors and urban birth were smaller for both outcomes (3–5%), as were childhood infections for BD. Urban residence had null (SCZ) or in the case of BD, negative PAFs with rural residence being the adverse risk factor. After inclusion of FGRS (adjusted model), there was slight attenuation in the PAF for adverse perinatal factors for SCZ, and the PAF for ACEs for both disorders.

### Sensitivity analyses

We examined the potential confounding effect of parental SCZ/BD, given that parental psychopathology may directly influence environmental exposures. Similar to the FGRS-adjusted models, there was minor attenuation of the ACEs estimates (Figure S4, Table S5).

Additional sensitivity analysis explored whether the effects of the environmental exposures on SCZ and BD exhibited significant modification across varying levels of familial risk. The multiplicative interactions were either statistically non-significant or weakly significant (i.e. *p* ≥ 0.01 and would not withstand adjustment for multiple testing) (Figure S5, Table S7). PAF estimates were robust across the adjusted and interaction models (Table S7). The similarity in the shape of the curves (Figure S5), along with the weak evidence for interaction effects provides stronger support that FGRS and exposures contribute independently to risk of SCZ and BD.

Finally, adjusting each of the PAF models for parental SES notably attenuated the estimates for ACEs and SCZ (exposure only PAF, 14.1%; SES adjusted PAF, 8.6%), but only slightly for BD (exposure only PAF, 19.8%; SES adjusted PAF, 17.2%) (Table S7).

## DISCUSSION

### Summary

Our novel investigations regarding the dose-response relationship with increasing degrees of FGRS revealed a strong relationship for both disorders, but SCZ risk was greater for those with highest FGRS. Both FGRS and environmental exposures contributed independently to risk of SCZ and BD: specifically, the risk associated with environmental exposures remained essentially unchanged when controlling for FGRS and vice versa. Regarding prevention, both ACEs and substance use – which exhibited the largest PAFs even after adjustment for FGRS - are promising targets. The findings provide insights into etiology, and the comparatively large PAFs for substance use and ACEs underscore their potential as viable targets for prevention.

### Comparison with prior studies

FGRS was robustly associated with cumulative increases in risk of SCZ and BD diagnosis. These findings are in agreement with and extend upon recent studies which report higher mean FGRS in SCZ and BD cases [5]. The risk associated with FGRS appears stronger for SCZ than for BD, especially at higher quintiles: those in the top 20% of FGRS risk had 9x higher risk of SCZ, compared to a 6x higher risk of BD; aside from substance use, this is distinctly higher than the risk conveyed by environmental exposures. A higher genetic risk for SCZ compared to BD aligns with observations in diagnoses among both parents and probands [13]. However, it remains unclear why this is the case, given that the heritability of these disorders is comparable [2–4].

No other investigations of FGRS have been conducted in the context of environmental risk which limits direct comparisons with existing literature. Our findings indicate that both FGRS and environmental exposures affect risk of SCZ and BD, with most environmental associations remaining virtually unchanged after adjustment for familial-genetic risk, except for a very modest attenuation of the risk associated with ACEs. This slight partial confounding of the association between ACEs and SCZ/BD by FGRS was also seen for parental psychiatric history, reflected by small decreases in IRR and in PAF under adjustment: as ACEs are entwined with parental behavior and psychological wellbeing, this confounding may indicate a degree of gene-environment correlation (rGE, i.e. genetically influenced differences in exposure to environmental risk factors). Similar to prior studies, we observed a higher prevalence of childhood trauma and abuse in those with a parental history of psychotic disorders (including SCZ and BD) [14–16]. Other genetically informed study designs report that while there is a degree of genetic confounding, it does not entirely explain the association between ACEs and SCZ/BD [17, 18]. Furthermore, Mendelian randomization studies find bidirectional causal effects of ACEs on SCZ, which could occur if parental psychosis traits affect maltreatment or genetic predisposition to SCZ influences the child’s behavior and the parent’s reactions [19].

High FGRS and substance use was associated with significantly elevated risks for SCZ and BD. The stability of these estimates across adjustment models is contrary to earlier findings in a Swedish co-relative study which reports substantial genetic confounding for substance use disorders [20]. This could be due to shared genetic risk for substance use disorders and SCZ/BD. However, a previous study examining FGRS for individuals diagnosed with major psychiatric disorders found that individuals diagnosed with substance use disorders had a comparatively lower mean FGRS for SCZ and BD, meaning that the occurrence of SCZ and BD in their family members is relatively less common compared to some other psychiatric disorders [5]. There is also a low-to-moderate genetic correlation (r = 0.18–0.35) between substance use traits with SCZ and BD [21]. Therefore, while there exists a degree of shared risk, the relationship between substance, genetic risk, and SCZ and BD, is likely more complex.

Among the limited high-quality studies available for comparison, a handful of Danish register studies demonstrate congruent findings for SCZ, but equivalent studies for BD are pending. Adjusting for family psychiatric history had negligible impact on the associations between several adverse perinatal factors and risk of SCZ [22]. Molecular-genetic studies (using polygenic risk scores, PRS, as an indicator of genetic liability) find independent effects of SCZ-PRS and a history of infections [23], and for birth in the most densely populated urban area on SCZ risk [24]. Yet, higher genetic liability for SCZ and BD is associated with urban birthplace or residence [25, 26]. We also observed a higher prevalence of urban birth/residence in those with higher FGRS, particularly for BD, but this was evident in both cases and controls. This is consistent with prior findings and suggests that selective local migration occurs in individuals with high genetic risk regardless of psychiatric diagnosis [25].

Our supplementary analyses demonstrate that findings were robust when substituting parental SCZ/BD with FGRS. The multiplicative interactions were either statistically non-significant or only weakly significant. Furthermore, the effect sizes and patterns of these interactions were also weak, indicating at best minor modification of a model where both environmental exposures and FGRS contribute to disease risk mostly independently. Our findings align with null results from a molecular genetic study (PRS) regarding the lack of interactions between infections and FGRS and risk of SCZ [23], and a lack of interactions between perinatal factors and family history on SCZ risk in a Danish register study [22]. Given the substantial magnitude of our sample exceeding 3000 SCZ cases and the inherent limitations in identifying interactions, it is likely that prior studies have been underpowered to detect interactions [1]. An interesting finding was the notable reduction in the PAF for ACEs for SCZ after adjusting for childhood SES, whereas the estimate for ACEs and BD remained largely unchanged, suggesting that the relationship between ACEs and SES may differ between the disorders.

Our main effect models indicate that for individuals who have both a high FGRS and carry a high-risk exposure like ACE or substance use, a (counterfactual) removal of the environmental exposure may reduce but cannot eliminate, the risk conveyed by their family background; in this setting, a high FGRS establishes a high baseline risk of SCZ or BD, which is modified by the pattern of non-familial exposures present in an individual. Still, ACEs and substance use, which individually accounted for up to 20% of SCZ/BD cases, represent important targets for prevention. In practice, however, it is not straightforward to entirely eliminate these exposures, which are unlikely to be purely environmental. Estimating PAFs assumes a causal relationship between the exposures and outcomes, and therefore PAFs likely overestimate the true impact. Nonetheless, given the magnitude PAFs associated with ACEs and substance use, which persisted following adjustment for FGRS, even modest reductions in their prevalence could lead to meaningful clinical impact. Furthermore, multi-pronged approaches addressing both individual and structural factors would be necessary to effectively mitigate these exposures.

### Strengths and limitations

The strengths of our study are the high-quality data from Swedish national registers, resulting in a large sample with many cases, multi-generational family histories, and rich exposure data. The validity of the FGRS score is contingent on the quality of the psychiatric diagnoses in these registers, which has been well-demonstrated for SCZ and BD [27–29]. However, our findings should be considered in the context of several limitations. First, register-based data only include a subset of all parents and offspring with substance misuse. In addition to diagnosed disorders, we included alcohol and drug-related criminal convictions to widen the definition of substance misuse, but our results are best interpreted as pertaining to more severe substance use problems in parents and offspring. Furthermore, while the FGRS calculation addresses several key considerations, other unaccounted for influential factors include survivor bias, and the lower likelihood of individuals with BD, and particularly SCZ, having children [30]. The FGRS calculation adjusts for cohabitation effects, however there is inherent complexity in disentangling the genetic and environmental influences within familial risk. It is unavoidable that for rare disorders there will be few affected relatives, which generates a skewed FGRS distribution. In our study, we utilized the entire sample to calculate FGRS under the assumption that the genetic liability would be present in the proband from birth. However, if these scores were intended for clinical predictive purposes, it would be necessary limit inclusion to family members diagnosed before the proband. We use objective measures including hospital contacts to assess our exposures, which will minimize recall bias and subjectivity, yet these likely capture more severe forms of substance use, infections and abuse as well as the potential for some degree of underreporting. We employed binary exposure measures as a pragmatic decision, but these may not fully capture the exposure’s complexity and subtleties, particularly for urbanicity [8]. Correspondingly, SCZ and BD are highly heterogeneous in terms of symptoms and clinical course, which cannot be fully captured via these register data. Additionally, the range of correlated analyses conducted preclude a clear approach for multiple testing correction; therefore, marginally significant *p*-values should be interpreted with caution. Future studies incorporating more comprehensive and nuanced measures, although more complex to model and interpret, could yield valuable insights.

## CONCLUSIONS

Environmental exposures and familial risk jointly contribute to risk of SCZ and BD. Familial genetic risk and molecular genetic risk demonstrate low correlations and appear to capture independent and complimentary aspects of SCZ and BD risk [31, 32], therefore integration of both, along with environmental risk factors may provide a more comprehensive understanding of these disorders.

Given that genetic risk is unmodifiable, prevention efforts may be best focused on ACEs and substance use. These findings highlight the importance of concurrently investigating genetic and environmental factors and provide new insights into SCZ and BD etiology.

## Supplementary Material

Supplement

**Supplementary information** The online version contains supplementary material available at https://doi.org/10.1038/s41380-026-03715-z.

## Figures and Tables

**Table 1.** Demographic and family charactertistics of the SCZ and BD matched samples.

Characteristic	SCZ matched sample			Characteristic	BD matched sample		
	Overall *N* = 20589	Control *N* = 17,532	Case *N* = 3057		Overall *N* = 93,669	Control *N* = 78,640	Case *N* = 15,029
**Sex**				**Sex**			
*Male*	13,674 (66%)	11,667 (67%)	2007 (66%)	*Male*	31,672 (34%)	26,605 (34%)	5067 (34%)
*Female*	6915 (34%)	5865 (33%)	1050 (34%)	*Female*	61,997 (66%)	52,035 (66%)	9962 (66%)
**FGRS quintile**				**FGRS quintile**			
*No affected relatives*	19,038 (92%)	16,528 (94%)	2510 (82%)	*No affected relatives*	79,150 (84%)	68,441 (87%)	10,709 (71%)
Q1	311 (1.5%)	256 (1.5%)	55 (1.8%)	Q1	2904 (3.1%)	2366 (3.0%)	538 (3.6%)
Q2	310 (1.5%)	224 (1.3%)	86 (2.8%)	Q2	2904 (3.1%)	2312 (2.9%)	592 (3.9%)
Q3	310 (1.5%)	210 (1.2%)	100 (3.3%)	Q3	2904 (3.1%)	2119 (2.7%)	784 (5.2%)
Q4	310 (1.5%)	184 (1.0%)	126 (4.1%)	Q4	2904 (3.1%)	1.912 (2.4%)	992 (6.6%)
Q5	310 (1.5%)	130 (0.7%)	180 (5.9%)	Q5	2904 (3.1%)	1490 (1.9%)	1414 (9.4%)
**Parent diagnosed with SCZ**				**Parent diagnosed with BD**			
*No parent with SCZ*	20,352 (99%)	17,444 (99%)	2908 (95%)	*No parent with BD*	90,879 (97%)	77,320 (98%)	13,559 (90%)
*Either parent*	237 (1.2%)	88 (0.5%)	149 (4.9%)	*Either parent*	2790 (3.0%)	1320 (1.7%)	1470 (9.8%)
**Total number of relatives (mean/sd) SCZ relatives (mean / sd)**	22.0 (9.6)	22.1 (9.5)	22.4 (10.2)	**Total number of relatives (mean/sd) BD relatives (mean / sd)**	22.4 (10.1)	22.4 (10.2)	22.0 (9.3)
	0.09 (0.33)	0.07 (0.28)	0.22 (0.53)		0.20 (0.54)	0.17 (0.48)	0.40 (0.74)

**Table 2.** Definitions of the early life exposure variables.

Exposure	Definition	Exposure timing	Register
Adverse Childhood Experiences (ACEs)	Any recorded ACE (consisting of parental separation, parental incarceration, parental death, parental substance use or child abuse)	Age 0–15	NPR, LISA, censuses, death, crime and suspect registers
Substance use	Diagnosis of substance abuse for any illicit substance or alcohol or a recorded drug-related crime	At least two years before SCZ or BD diagnosis	NPR, crime and suspect registers
Childhood infections	Any infection, including gastrointestinal, skin, genitourinary and respiratory infections, septicemia, viral hepatitis, and infections associated with SCZ/BD (*T.gondii*, CMV, Herpes, CNS)	Age 0–15	NPR
Adverse perinatal factors	Any recorded perinatal factors from the validated Lewis-Murray obstetric complications scale [36] plus additional pregnancy and perinatal factors associated with SCZ/BD (small head circumference < 32 cm, gestational diabetes, jaundice, low APGAR score, high parity (3 + previous pregnancies)) [10, 12]	Birth	NPR, MBR
Urban birth and urban longest residence	Population density category of place of birth and longest residence (rural or urban)	Birth, and before SCZ/BD diagnosis	LISA, Parish

Full descriptions of the variable definitions can be found in the supplementary methods. The variables were derived from several registers including the National Patient Register (NPR) which contains data on all inpatient diagnoses since 1973 and outpatient data since 2001; the Longitudinal integrated database for health insurance and labour market studies (LISA) databases and the Censuses (1960–90) contain information on family structure and socioeconomic factors; death register contains date of death; and the National Crime Register includes all criminal convictions since 1973.

**Table 3.** IRR for exposures with models unadjusted and adjusted for FGRS.

SCZ	Crude			Adjusted		
Exposure	IRR	95%CI	p	IRR	95%CI	p
Any ACE	**1.65**	(1.52–1.78)	<0.001	**1.54**	(1.42–1.67)	<0.001
Substance use	**6.21**	(5.45–7.07)	<0.001	**6.14**	(5.38–7.02)	<0.001
Childhood infection	1.10	(1.00–1.21)	0.053	1.09	(0.98–1.20)	0.102
Adverse perinatal factor	**1.13**	(1.04–1.22)	0.004	**1.10**	(1.02–1.20)	0.019
Urban birth	**1.32**	(1.13–1.54)	<0.001	**1.36**	(1.16–1.59)	<0.001
Urban residence	**1.21**	(1.07–1.38)	0.004	**1.24**	(1.09–1.42)	0.002
BD	Crude			Adjusted		
Exposure	IRR	95%CI	p	IRR	95%CI	p
Any ACE	**1.74**	(1.68–1.80)	<0.001	**1.64**	(1.58–1.71)	<0.001
Substance use	**4.84**	(4.56–5.14)	<0.001	**4.78**	(4.49–5.08)	<0.001
Childhood infection	**1.30**	(1.24–1.35)	<0.001	**1.28**	(1.23–1.34)	<0.001
Adverse perinatal factor	**1.10**	(1.06–1.14)	<0.001	**1.10**	(1.06–1.14)	<0.001
Urban birth	1.02	(0.96–1.10)	0.502	1.01	(0.94–1.08)	0.867
Urban residence	**0.90**	(0.85–0.95)	<0.001	**0.90**	(0.84–0.95)	<0.001

Crude (Unadjusted) models estimate the IRR associated with the individual exposures. Adjusted models are the individual exposure models adjusted for SCZ or BD FGRS. Estimates are relative to unexposed. Bold indicates *p* < 0.05.

## Data Availability

The data used in this study are available through application to the respective register holders in Sweden.

## CODE AVAILABILITY

The cohort generation (case-control matching) and statistical analyses were conducted in R (version 4.2.3) [33], using the FGRS package [34] to calculate FGRS, the survival package (version 3.2–10) for regression analyses [35], and package AF, to calculate the PAF [12].
