# Supplementary material for: Effects of family genetic risk scores and environmental factors on risk of schizophrenia and bipolar disorder
Source: Mol Psychiatry. 2026 Jul 3;31(11):6456–63. doi: 10.1038/s41380-026-03715-z (PMC13428273; doi:10.1038/s41380-026-03715-z)
Supplement: Supplementary file 1 [file 41380_2026_3715_MOESM1_ESM.docx]

# Supplementary Methods

## ICD diagnostic codes for outcomes schizophrenia and bipolar disorder

*International Classification of Diseases, Ninth Revision* (ICD-9, 1987-1996), and *International Statistical Classification of Diseases and Related Health Problems, Tenth Revision* (ICD-10, 1997-present) codes were used for defining outcomes.

SCZ was defined as ICD-9 295 and ICD-10 F20 with latent (ICD-9 295F), simple (ICD-9 295A; ICD-10 F20.6), acute (ICD-9 295E), post-schizophrenic depression (ICD-10 F20.4), and schizoaffective disorders (ICD-9 295H) excluded.

BD was defined as ICD-9 296 with unipolar aﬀective psychosis, melancholic form (ICD-9 296B), other (ICD-9 296W) and unspecified (ICD-9 296X) excluded, and all ICD-10 F30 and F31.

## Coding environmental exposures using Swedish registry data

Table S1. Coding Individual Exposure variables

| **Exposure coding from register variables** | | | | |
| --- | --- | --- | --- | --- |
| **Exposure group** | **Sub-exposure** | **Register(s)** | **Register years** | **Variable coding** |
| ACEs | Parental separation | Census | 1975, 1980, 1985, 1990 | Separated: If the parents divorced/separated between ages 0-15.  Not separated: the parents are married or cohabitating ages 0-15.  Coded as missing if the mother was single (at birth) or if a parent died when the individual was aged 0-15 |
|  |  | Longitudinal integrated database for health insurance and labour market studies (LISA) | 1990+ |  |
|  |  | Medical birth register | 1973+ |  |
|  | Child abuse (physical, sexual, neglect) | Crime register | 1965+ | Coded if either parent was convicted of abusing their offspring under the law 'Sexual intercourse with offspring' (1965+). |
|  | Parental substance abuse (drug abuse, alcohol abuse, drunk/drug driving, drug-related crime) | Crime register | 1965+ | Driving under the influence of drugs or alcohol (1951+). Drug possession (1968+). |
|  |  | Suspect register | 1975+ | Drug penalty law, holding, use, holding and use (1998+).  Drunk driving, aggravated drunk driving under the influence of alcohol alone or under the influence of both alcohol and drugs (1975+). Driving under the influence of drugs only (2000+) |
|  | Parental incarceration | Sanction register | 1973+ | If either parent spent time in prison when the individual was aged 0-15 |
|  | Parental death | Death register | 1961+ | If either parent died when the individual was aged 0-15 |
|  | Violence in the home (domestic violence) | National Crime Register | 1973+ | Gross violation of integrity and gross violation of a women’s integrity (1998+). |
|  |  | Suspect register | 1975+ | Serious violation of women's rights (1998+). Abuse, not rough or rough, indoor against woman/man 18 years of age or older in close relationship (i.e. been married, cohabits, or cohabited, or has common children) (2008+). |
| Substance use  (proband) | Drunk/drug driving, drug-related crime | Crime register | 1965+ | Driving under the influence of drugs or alcohol (1951+). Drug possession (1968+). |
| Adverse perinatal factors |  | Medical birth register | 1973+ | twin/multiple birth, gestational age <37 or >42 weeks, caesarean delivery, malpresentation (breech/abnormal presentation), instrument (forceps/vacuum) delivery, hypoxia-anoxia, low birth weight (<2.5kg, LBW) or a congenital malformation, low APGAR score, high parity (3+ previous pregnancies), small head circumference <32cm. |

| **ICD codes from registers** | | | | | |
| --- | --- | --- | --- | --- | --- |
| **Exposure group** | **Sub-exposure** | **Register** | **ICD8** | **ICD9** | **ICD10** |
| ACEs | Child abuse (violence, abuse, or neglect) | National patient register | 994.4, 994.98, 996.88, 996.99, 994.2, 994.3, 994.4, (E960 - E969) | 995F, 994X, 994X, 994C, 994D, 994E, 994F, E960-E969 | T73, T74, X85-Y09 |
|  | Parental substance abuse (ACEs) | National patient register | 303, 304 | 303, 304, 305 | F10-F16, F18, F19 |
| Substance use  (proband) | Diagnoses of substance use disorders | National patient register | 303, 304 | 303, 304, 305 | F10-F16, F18, F19 |
| Childhood infections | Infections (0-15 years) | National patient register | 000-014, 016, 017.01-017.09, 020.10, 027.01, 036.00, 038.00-038.99, 039.92, 040.00-043.99, 045.00-046.99, 050-057, 062.00-065.99, 70, 071.99, 072, 075.02, 079.5, 079.20, 084.00, 090-099, 094.00-094.98, 110-111, 127.99, 130, 320.00-320.80, 320.88-320.99, 322.00-322.03, 392.99,460.99, 461.00-461.09, 462.01, 462.02, 462.09, 463.01, 463.09, 464.01-464.09, 465.99, 466.99, 470-474, 480.99, 481.99-482.98, 483.99-486.09, 490.99-491.09, 501.99, 502.00-503.09, 508.00-508.03, 510.01-510.09, 511.10, 513.99, 519.92, 522.50, 527.30, 528.00, 528.30, 540.00-540.99, 562.00, 562.19, 566.00-566.01, 567.00-567.02, 569.00, 572.99, 577. 01, 590.00-590.99, 595.00-595.02, 597.00, 599.02, 601.00, 604.00, 604.01, 607.30, 611.00, 611.01, 612.01-614.99, 616.00-616.03, 620.00-620.99, 622.00-622.19, 629.40, 630.00-630.09, 635.00-635.99, 680.00-680.90, 681.00-682.99, 684.00-684.09, 686.00-686.98, 761.2, 761.4, 782.9 | 001-012W, 013-X, 014, 016, 017A, 031A, 031B, 033-034B, 036A-B, 038A-X, 045-049X, 050-057, 052A, 052B, 053A, 054, 055A, 055B, 056A, 070A-X 071, 072, 074D, 078F , 090, 090E, 091D, 091-098, 094-X, 099, 110-111, 112D, 112E, 112B-C, 122B, 123, 127, 129, 130, 131A, 136E-F, 320-X, 321A, 321B-H, 321W, 323A, 323C-D, 324-X, 392-X, 460-465, 466, 466-B, 473-X, 475, 480-X, 481-482X, 483, 485, 486, 487 , 490, 491B, 510-X, 511B, 513-B, 522E, 522H, 526E, 527D, 528A, 528D, 540A-X, 562-B, 566, 567-C, 569F, 575A,590-X, 595-X, 597A, 597W, 599A, 601-D, 603B, 604A, 604X, 607B-C, 608A-E, 611A, 614-X, 615A-X, 616-X, 646F, 646G, 646W, 674D 680A 681-682X, 683, 684, 685-686X, 760C, 771B, 771C , 785F | A00-18.1,  A18.4, A20.0, A20.2, A20.3, A21.2, A22.1, A22.0, A22.8, A26.0, A31.0, A31.1, A32, A32.1, A36.3, A37, A38, A39.0, A40-A41, A48.1, A50-64, A70-74, A80-89, B00-B09, B15-B19, B25-B27, B35-36, B37.1, B37.2, B37.3-37.4, B37.5, B38.4, B39-42, B43.0, B43.1, B43.2, B44, B45.0, B45.1, B45.2, B46.0, B46.1, B46.3, B50.0, B55.1, B57.4, B58, B59, B60.2, B68, B69, B69.0, B70, B71, B77-B82, B83.2, B83.8, G00-00.9, G01, G02.0-02.8, G04, G04.2, G04.9, G05.0, G05.1, G05.2, G06.0-6.2, G07, I02-02.9,J00-J22, J32, J34.0, J35.0, J36, J37, J39.0-39.1, J40-42,K04.4-K04.7, K10.2, K11.3, K12.1, K12.2, K35, K57, K61, K63.0, K65, K81, L00-L08, L30.3, L70.0, M02.1, N10-12, N13.6, N15.1, N15.9, N30-30.3, N30.8-30.9, N34-34.1, N39.0, N41-41.3, N43.1, N45.0-45.9, N48.1-48.2, N49-49.9, N61, N70-76.8, N98.0, O23, O26.4, O85-O86, P00.2, P35.1, P35.2, P37.1, T62.9 |
| **Obstetric complications** | Neonatal jaundice | Medical birth register diagnoses | 774, 778.93 | 773-774 | P57-P59 |
|  | Rubella/Syphilis |  | 761.3, 056, 090-097 | 771.0, 056, 090-097 | P350, B06, A50-A53 |
|  | Rhesus incompatibility |  | 774.0, 775.0 | 773A | P550 |
|  | Severe pre-eclampsia |  | 637 | 642.5 | O141 |
|  | Incubator (anoxia/hypoxia) |  | 764.4,765.4,766.4, 767.4, 768.4,776 | 768C-768X, 770WW,775WB | P84, P20-21 |
|  | Gestational diabetes |  | 7611 | 648A | O24 |

To determine the age of the child when the crime-related ACE happened, the crime start date was used. If the start date of the crime date was missing, then the decision date was used. If there is no report of a specific ACE occurring then these are set to ‘No' i.e. if there is no reported abuse, assume that no abuse happened. In Swedish ICD9 codes there are no subcategories for non-dependent drug abuse. Obstetric complications are derived from the validated Lewis-Murray OC scale[1], plus additional pregnancy and perinatal factors associated with SCZ/BD[2, 3]. There were several Lewis-Murray OCs which were not possible to include due to lack of ICD codes or data were not collected. These included: antepartum haemorrhage, premature rupture of membranes, cord prolapse or knotted, and long labour. There was overlap between neonatal jaundice and rhesus ICD codes (ICD-8 774.0, ICD-9 773A), although as rhesus was a rare exposure (0.1%) this affected few cases.

Urbanicity was determined from population size per municipality (kommun), using data from Statistics Sweden (1968-2013)[4]. Sweden is currently divided into 290 administrative municipalities. For each year, we determined the population density as the number of inhabitants divided by the area of municipality in km^2^. The urbanicity category for each municipality was assigned according to the Eurostat definitions:

- High-density: a density of at least 1500 inhabitants per km^2^ and a minimum population of 50,000
- Urban: a density of at least 300 inhabitants per km^2^ and a minimum population of 5,000
- Rural: a density less than 300 inhabitants per km^2^

For simplicity, high density and urban were combined. The longest residence for each individual was the most frequent urbanicity category. If someone spent equivalent time in two categories, they were assigned the higher risk category (i.e. urban/high-density>rural). Each participant was classified based on population density of their municipality (i) at birth, and (ii) of longest residence prior to SCZ/BD diagnosis or sampling date. Urbanicity at birth and of longest residence were analysed as binary variables (urban vs rural).

Socioeconomic status (SES) was determined from the highest achieved parental education, and highest parental disposable income when the child was age 16. SES variables were taken from either the Labour statistics based on administrative sources 1985-1989, or from the longitudinal integrated database for health insurance and labour market studies (LISA,1990+)[5]. Highest parental education, which utilizes the SUN2000 nomenclature (Swedish education nomenclature), was extracted when the child was aged 16. Education for both parents was categorized by compulsory, secondary, higher and postgraduate, and the highest achieved parental education was the highest level from either the biological mother or father (Table S4).

Table S2. Categorising education level in LISA

| Code SUN2000NIVA (OLD) | Code SUN2000NIVA (new) | Category | Years schooling |
| --- | --- | --- | --- |
| 1, 2 | 1  2 | Compulsory (i.e. Primary and lower secondary education) | 0-9 years (compulsory schooling) |
| 3, 4 | 3 | Upper secondary | 0-3 years upper secondary education |
| 5 | 4-5.3 | Higher education (i.e. university) | 0-3 years post-secondary education |
| 6, 7 | 5.4-6 | Postgraduate | 4 years post-secondary education |

Household disposable income from LISA 1990-2013 (DISPINKFAM) was determined for both parents for the years when the individual was aged 14-16. Household income was averaged (mean) from data from the three years, and the highest income (from mother/father) was stratified into quartiles based on income by year data from the controls. Due to data only being available from 1990 onwards, those born in 1973 will be missing parental income data, those born in 1974 only have data from the year they are age 16, and those born in 1975 only have data at ages 15-16.

# Supplementary RESULTS

Table S3 Prevalence of exposure to environmental factors across FGRS quintiles and by parental SCZ in SCZ matched sample

| **SCZ matched sample** | FGRS quintiles | | | | | | | | | | | |  | Parent diagnosed with SCZ | | | |
| --- | --- | --- | --- | --- | --- | --- | --- | --- | --- | --- | --- | --- | --- | --- | --- | --- | --- |
|  | **Ref** | | **Q1** | | **Q2** | | **Q3** | | **Q4** | | **Q5** | |  | **No parent SCZ** | | **Either parent** | |
| **Exposure** | case,  N = 2,510 | ctrl,  N = 16,528 | case,  N = 55 | ctrl,  N = 256 | case,  N = 86 | ctrl,  N = 224 | case,  N = 100 | ctrl,  N = 210 | case, N = 126 | ctrl,  N = 184 | case,  N = 180 | ctrl,  N = 130 |  | case,  N = 2,908 | ctrl,  N = 17,444 | case,  N = 149 | ctrl,  N = 88 |
| Any ACE | 1,224 (49%) | 6,398 (39%) | 33 (60%) | 101 (39%) | 48 (56%) | 99 (44%) | 70 (70%) | 97 (46%) | 78 (62%) | 87 (47%) | 118 (66%) | 76 (58%) |  | 1,451 (50%) | 6,790 (39%) | 120 (81%) | 68 (77%) |
| SUD | 543 (22%) | 755 (4.6%) | 21 (38%) | 15 (5.9%) | 17 (20%) | 18 (8.0%) | 23 (23%) | 14 (6.7%) | 25 (20%) | 10 (5.4%) | 33 (18%) | 5 (3.8%) |  | 630 (22%) | 814 (4.7%) | 32 (21%) | - |
| Childhood infection | 562 (22%) | 3,456 (21%) | 14 (25%) | 49 (19%) | 16 (19%) | 49 (22%) | 25 (25%) | 54 (26%) | 32 (25%) | 36 (20%) | 45 (25%) | 30 (23%) |  | 659 (23%) | 3,660 (21%) | 35 (23%) | 14 (16%) |
| Adverse perinatal factor | 1,124 (45%) | 7,036 (43%) | 30 (56%) | 122 (48%) | 43 (52%) | 102 (47%) | 46 (47%) | 93 (44%) | 69 (56%) | 81 (45%) | 81 (45%) | 58 (45%) |  | 1,319 (46%) | 7,451 (43%) | 74 (51%) | 41 (47%) |
| Urban birth | 575 (25%) | 3,641 (24%) | 13 (25%) | 34 (15%) | 14 (18%) | 39 (19%) | 20 (22%) | 55 (29%) | 28 (25%) | 39 (23%) | 38 (23%) | 40 (36%) |  | 662 (25%) | 3,831 (24%) | 26 (19%) | 17 (23%) |
| Urban residence | 549 (22%) | 3,503 (21%) | 12 (22%) | 37 (14%) | 16 (19%) | 39 (17%) | 18 (18%) | 41 (20%) | 31 (25%) | 43 (23%) | 35 (19%) | 38 (29%) |  | 633 (22%) | 3,685 (21%) | 28 (19%) | 16 (18%) |

Cells containing values <5 individuals removed to preserve anonymity.

Table S4 PREVALENCE OF EXPOSURE TO ENVIRONMENTAL FACTORS ACROSS FGRS QUINTILES AND BY PARENTAL BD in BD matched sample

| **BD matched sample** | FGRS quintiles | | | | | | | | | | | |  | Parent diagnosed with BD | | | |
| --- | --- | --- | --- | --- | --- | --- | --- | --- | --- | --- | --- | --- | --- | --- | --- | --- | --- |
|  | **Ref** | | **Q1** | | **Q2** | | **Q3** | | **Q4** | | **Q5** | |  | **No parent BD** | | **Either parent** | |
| **Exposure** | case,  N = 10,709 | ctrl,  N = 68,441 | case,  N = 538 | ctrl,  N = 2,366 | case,  N = 592 | ctrl,  N = 2,312 | case,  N = 784 | ctrl,  N = 2,119 | case,  N = 992 | ctrl,  N = 1,912 | case,  N = 1,414 | ctrl,  N = 1,490 |  | case,  N = 13,559 | ctrl,  N = 77,320 | case,  N = 1,470 | ctrl,  N = 1,320 |
| Any ACE | 5,575 (52%) | 27,219 (40%) | 323 (60%) | 1,044 (44%) | 324 (55%) | 1,059 (46%) | 466 (59%) | 1,041 (49%) | 564 (57%) | 905 (47%) | 861 (61%) | 785 (53%) |  | 7,144 (53%) | 31,231 (40%) | 969 (66%) | 822 (62%) |
| SUD | 1,832 (17%) | 2,814 (4.1%) | 108 (20%) | 135 (5.7%) | 117 (20%) | 111 (4.8%) | 136 (17%) | 112 (5.3%) | 156 (16%) | 103 (5.4%) | 209 (15%) | 87 (5.8%) |  | 2,320 (17%) | 3,273 (4.2%) | 238 (16%) | 89 (6.7%) |
| Childhood infection | 2,694 (25%) | 14,157 (21%) | 123 (23%) | 524 (22%) | 131 (22%) | 488 (21%) | 223 (28%) | 479 (23%) | 240 (24%) | 415 (22%) | 397 (28%) | 319 (21%) |  | 3,427 (25%) | 16,071 (21%) | 381 (26%) | 311 (24%) |
| Adverse perinatal factor | 4,563 (43%) | 27,898 (41%) | 268 (50%) | 1,014 (43%) | 253 (43%) | 984 (43%) | 355 (46%) | 876 (42%) | 401 (41%) | 775 (41%) | 626 (45%) | 584 (39%) |  | 5,835 (43%) | 31,588 (41%) | 631 (43%) | 543 (41%) |
| Urban birth | 2,295 (23%) | 14,189 (22%) | 64 (13%) | 353 (16%) | 95 (17%) | 426 (19%) | 141 (19%) | 431 (21%) | 233 (24%) | 457 (25%) | 327 (24%) | 358 (25%) |  | 2,816 (22%) | 15,900 (22%) | 339 (24%) | 314 (25%) |
| Urban residence | 2,003 (19%) | 13,027 (19%) | 53 (9.9%) | 316 (13%) | 76 (13%) | 376 (16%) | 117 (15%) | 388 (18%) | 192 (19%) | 410 (21%) | 263 (19%) | 321 (22%) |  | 2,417 (18%) | 14,559 (19%) | 287 (20%) | 279 (21%) |

Table S5 IRR for exposures, with models unadjusted and adjusted for family risk (FGRS, parental SCZ/BD)

| **SCZ** | Unadjusted | | |  | Exposures adjusted for FGRS | | |  | Exposures adjusted for parental SCZ | | |
| --- | --- | --- | --- | --- | --- | --- | --- | --- | --- | --- | --- |
| Exposure | IRR | 95%CI | p |  | IRR | 95%CI | p |  | IRR | 95%CI | p |
| Any ACE | **1.65** | (1.52-1.78) | <0.001 |  | **1.54** | (1.42- 1.67) | <0.001 |  | **1.55** | (1.43- 1.68) | <0.001 |
| SUD | **6.21** | (5.45-7.07) | <0.001 |  | **6.14** | (5.38- 7.02) | <0.001 |  | **6.16** | (5.40- 7.02) | <0.001 |
| Childhood infection | 1.10 | (1.00-1.21) | 0.053 |  | 1.09 | (0.98- 1.20) | 0.102 |  | 1.10 | (1.00- 1.21) | 0.06 |
| Adverse perinatal factor | **1.13** | (1.04-1.22) | 0.004 |  | **1.10** | (1.02- 1.20) | 0.019 |  | **1.11** | (1.03- 1.21) | 0.01 |
| Urban birth | **1.32** | (1.13-1.54) | <0.001 |  | **1.36** | (1.16- 1.59) | <0.001 |  | **1.36** | (1.16- 1.59) | <0.001 |
| Urban residence | **1.21** | (1.07-1.38) | 0.004 |  | **1.24** | (1.09- 1.42) | 0.002 |  | **1.24** | (1.08- 1.41) | 0.002 |
| FGRS Q1 | **1.36** | (1.01- 1.85) | 0.046 |  |  |  |  |  |  |  |  |
| FGRS Q2 | **2.34** | (1.80- 3.05) | <0.001 |  |  |  |  |  |  |  |  |
| FGRS Q3 | **3.10** | (2.39- 4.01) | <0.001 |  |  |  |  |  |  |  |  |
| FGRS Q4 | **4.42** | (3.45- 5.68) | <0.001 |  |  |  |  |  |  |  |  |
| FGRS Q5 | **9.27** | (7.14-12.03) | <0.001 |  |  |  |  |  |  |  |  |
| Parental SCZ | **9.63** | (7.17-12.94) | <0.001 |  |  |  |  |  |  |  |  |
|  |  |  |  |  |  |  |  |  |  |  |  |
| **BD** | Unadjusted | | |  | Exposures adjusted for FGRS | | |  | Exposures adjusted for parental BD | | |
| Exposure | IRR | 95%CI | p |  | IRR | 95%CI | p |  | IRR | 95%CI | p |
| Any ACE | **1.74** | (1.68-1.80) | <0.001 |  | **1.64** | (1.58-1.71) | <0.001 |  | **1.65** | (1.59-1.71) | <0.001 |
| SUD | **4.84** | (4.56-5.14) | <0.001 |  | **4.78** | (4.49-5.08) | <0.001 |  | **4.80** | (4.51-5.10) | <0.001 |
| Childhood infection | **1.30** | (1.24-1.35) | <0.001 |  | **1.28** | (1.23-1.34) | <0.001 |  | **1.29** | (1.23-1.35) | <0.001 |
| Adverse perinatal factor | **1.10** | (1.06-1.14) | <0.001 |  | **1.10** | (1.06-1.14) | <0.001 |  | **1.10** | (1.06-1.14) | <0.001 |
| Urban birth | 1.02 | (0.96-1.10) | 0.502 |  | 1.01 | (0.94-1.08) | 0.867 |  | 1.02 | (0.95-1.10) | 0.532 |
| Urban residence | **0.90** | (0.85-0.95) | <0.001 |  | **0.90** | (0.84-0.95) | <0.001 |  | **0.90** | (0.84-0.95) | <0.001 |
| FGRS Q1 | **1.44** | (1.30-1.59) | <0.001 |  |  |  |  |  |  |  |  |
| FGRS Q2 | **1.65** | (1.50-1.82) | <0.001 |  |  |  |  |  |  |  |  |
| FGRS Q3 | **2.31** | (2.11-2.52) | <0.001 |  |  |  |  |  |  |  |  |
| FGRS Q4 | **3.26** | (3.00-3.55) | <0.001 |  |  |  |  |  |  |  |  |
| FGRS Q5 | **6.10** | (5.62-6.62) | <0.001 |  |  |  |  |  |  |  |  |
| Parental BD | **6.30** | (5.80-6.84) | <0.001 |  |  |  |  |  |  |  |  |

Unadjusted models estimate the IRR associated for the individual exposures, or the categorical FGRS quintiles, or the binary affected parent measure (either parent diagnosed with SCZ/BD, sensitivity analysis). Adjusted models are the individual exposure models adjusted for FGRS or parental SCZ/BD. Estimates are relative to unexposed, or no affected relatives (FGRS), or no affected parents (parental SCZ/BD). Bold indicates p<0.05.

Table s6 Wald test for differences in FGRS estimates between SCZ and BD

| Exposure | SCZ IRR | BD IRR | IRR ratio | 95%CI | p |
| --- | --- | --- | --- | --- | --- |
| FGRS Q1 | **1.36** | **1.44** | 0.95 | (0.69-1.31) | 0.742 |
| FGRS Q2 | **2.34** | **1.65** | 1.42 | (1.07-1.88) | **0.015** |
| FGRS Q3 | **3.10** | **2.31** | 1.34 | (1.02-1.76) | **0.034** |
| FGRS Q4 | **4.42** | **3.26** | 1.36 | (1.04-1.76) | **0.024** |
| FGRS Q5 | **9.27** | **6.10** | 1.52 | (1.16-2.00) | **0.003** |

Bold indicates p<0.05.

Table S7 Population attributable fractions (PAF) for each of the estimated models

| **SCZ** | Exposure only | | Exposure + FGRS | | Exposure * FGRS | |
| --- | --- | --- | --- | --- | --- | --- |
| **UNADJUSTED** | **PAF** | **95%CI** | **PAF** | **95%CI** | **PAF** | **95%CI** |
| ACE | 14.1 | (10.6,17.6) | 11.8 | (8.0,15.5) | 12.0 | (8.0,15.9) |
| SUD | 18.3 | (17.6,19.0) | 18.3 | (17.6,19.0) | 18.1 | (17.3,18.9) |
| Childhood infection | 1.2 | (-1.1,3.5) | 0.9 | (-1.5,3.3) | 1.2 | (-1.2,3.6) |
| Adverse perinatal factors | 4.5 | (0.8,8.1) | 3.6 | (-0.2,7.4) | 2.6 | (-1.7,6.9) |
| Urban birth | 4.3 | (0.0,8.5) | 4.9 | (0.6,9.1) | 4.2 | (-0.2,8.7) |
| Urban residence | 0.1 | (-3.8,3.9) | 0.2 | (-3.7,4.2) | -0.3 | (-4.5,3.8) |
|  |  |  |  |  |  |  |
| **ADJUSTED FOR SES** | **PAF** | **95%CI** | **PAF** | **95%CI** | **PAF** | **95%CI** |
| ACE | 8.6 | (4.0,13.1) | 7.1 | (2.3,11.8) | 6.6 | (1.3,11.9) |
| SUD | 18.1 | (17.4,18.9) | 18.1 | (17.4,18.9) | 17.9 | (17.1,18.7) |
| Childhood infection | 1.6 | (-0.8,3.9) | 1.3 | (-1.2,3.7) | 1.4 | (-1.1,3.9) |
| Adverse perinatal factors | 3.6 | (-0.3,7.5) | 3.1 | (-0.9,7.1) | 1.9 | (-2.7,6.5) |
| Urban birth | 4.5 | (0.0,9.0) | 5.0 | (0.6,9.5) | 4.5 | (-0.1,9.2) |
| Urban residence | -0.4 | (-4.6,3.8) | -0.2 | (-4.5,4.0) | -0.6 | (-5.0,3.8) |
| **BD** | Exposure only | | Exposure + FGRS | | Exposure * FGRS | |
| **UNADJUSTED** | **PAF** | **95%CI** | **PAF** | **95%CI** | **PAF** | **95%CI** |
| ACE | 19.8 | (18.4,21.2) | 17.7 | (16.2,19.3) | 17.1 | (15.5,18.8) |
| SUD | 13.2 | (12.9,13.5) | 13.2 | (12.9,13.5) | 13.0 | (12.7,13.4) |
| Childhood infection | 5.0 | (4.0,5.9) | 4.9 | (3.9,5.9) | 5.0 | (4.0,6.0) |
| Adverse Perinatal Factors | 3.3 | (1.7,4.8) | 3.3 | (1.7,4.9) | 3.6 | (2.0,5.2) |
| Urban birth | 2.9 | (1.1,4.7) | 2.5 | (0.7,4.4) | 2.4 | (0.5,4.3) |
| Urban residence | -4.2 | (-6.1, -2.4) | -3.9 | (-5.8, -2.0) | -4.2 | (-6.1, -2.2) |
|  |  |  |  |  |  |  |
| **ADJUSTED FOR SES** | **PAF** | **95%CI** | **PAF** | **95%CI** | **PAF** | **95%CI** |
| ACE | 17.2 | (15.5,18.9) | 15.6 | (13.8,17.4) | 15.0 | (13.1,17.0) |
| SUD | 13.2 | (12.9,13.6) | 13.2 | (12.9,13.6) | 13.1 | (12.7,13.4) |
| Childhood infection | 5.0 | (4.0,6.0) | 5.0 | (4.0,6.0) | 5.0 | (4.0,6.1) |
| Adverse Perinatal Factors | 2.6 | (0.9,4.2) | 2.7 | (1.0,4.3) | 2.9 | (1.2,4.6) |
| Urban birth | 2.3 | (0.3,4.2) | 1.9 | (-0.1,3.9) | 1.8 | (-0.2,3.8) |
| Urban residence | -3.9 | (-5.8, -2.0) | -3.6 | (-5.5, -1.6) | -3.8 | (-5.8, -1.9) |

Sensitivity analysis included the interaction models (Exposure*FGRS) and SES adjusted models which included parental highest education (age 16) and household disposable income age 14-16 years as covariates.

Table S8 IRR FOR SCZ AND BD interaction models relative to no affected relatives and no exposure for each FGRS QUINTILe (sensitivity analysis).

|  |  | **SCZ** | | | | |  | **BD** | | | | |
| --- | --- | --- | --- | --- | --- | --- | --- | --- | --- | --- | --- | --- |
|  |  | **No exposure** | |  | **Exposure** | |  | **No exposure** | |  | **Exposure** | |
| **Exposure** | **Quintile** | **IRR** | **95%CI** |  | **IRR** | **95%CI** |  | **IRR** | **95%CI** |  | **IRR** | **95%CI** |
| ACE | Q1 | 1.03 | (0.65, 1.63) |  | 2.75 | (1.80, 4.21) |  | 1.29 | (1.11, 1.50) |  | 2.51 | (2.20, 2.87) |
|  | Q2 | 2.36 | (1.60, 3.47) |  | 3.21 | (2.23, 4.63) |  | 1.73 | (1.51, 1.99) |  | 2.56 | (2.24, 2.92) |
|  | Q3 | 1.98 | (1.30, 3.02) |  | 5.88 | (4.18, 8.27) |  | 2.34 | (2.05, 2.67) |  | 3.53 | (3.14, 3.98) |
|  | Q4 | 4.01 | (2.74, 5.87) |  | 6.51 | (4.68, 9.06) |  | 3.40 | (3.00, 3.84) |  | 4.98 | (4.43, 5.59) |
|  | Q5 | 9.15 | (6.04, 13.86) |  | 12.51 | (8.96, 17.48) |  | 6.65 | (5.87, 7.54) |  | 8.73 | (7.83, 9.74) |
| SUD | Q1 | 1.11 | (0.76, 1.61) |  | 12.63 | (6.02, 26.51) |  | 1.42 | (1.28, 1.59) |  | 5.83 | (4.44, 7.67) |
|  | Q2 | 2.50 | (1.86, 3.34) |  | 7.16 | (3.44, 14.93) |  | 1.63 | (1.47, 1.81) |  | 7.82 | (5.91, 10.36) |
|  | Q3 | 3.03 | (2.27, 4.03) |  | 13.36 | (6.18, 28.85) |  | 2.32 | (2.11, 2.55) |  | 8.94 | (6.81, 11.73) |
|  | Q4 | 4.59 | (3.49, 6.03) |  | 18.19 | (8.22, 40.25) |  | 3.34 | (3.05, 3.66) |  | 12.22 | (9.31, 16.04) |
|  | Q5 | 9.73 | (7.36, 12.86) |  | 40.51 | (15.52, 105.74) |  | 6.23 | (5.71, 6.80) |  | 20.76 | (15.57, 27.68) |
| Infections | Q1 | 1.28 | (0.90, 1.82) |  | 1.80 | (0.97, 3.34) |  | 1.48 | (1.33, 1.66) |  | 1.67 | (1.36, 2.05) |
|  | Q2 | 2.45 | (1.82, 3.29) |  | 2.16 | (1.19, 3.90) |  | 1.72 | (1.54, 1.92) |  | 1.88 | (1.54, 2.31) |
|  | Q3 | 3.24 | (2.40, 4.35) |  | 2.90 | (1.75, 4.79) |  | 2.24 | (2.02, 2.49) |  | 3.11 | (2.63, 3.68) |
|  | Q4 | 4.17 | (3.14, 5.55) |  | 5.84 | (3.47, 9.80) |  | 3.36 | (3.06, 3.70) |  | 3.84 | (3.23, 4.55) |
|  | Q5 | 8.85 | (6.60, 11.88) |  | 11.74 | (6.81, 20.26) |  | 5.90 | (5.37, 6.48) |  | 8.62 | (7.33, 10.15) |
| Adverse perinatal factors | Q1 | 1.20 | (0.76, 1.90) |  | 1.66 | (1.09, 2.52) |  | 1.32 | (1.15, 1.51) |  | 1.72 | (1.49, 1.98) |
|  | Q2 | 2.19 | (1.50, 3.21) |  | 2.70 | (1.84, 3.95) |  | 1.72 | (1.51, 1.95) |  | 1.71 | (1.48, 1.98) |
|  | Q3 | 2.91 | (2.05, 4.14) |  | 3.70 | (2.51, 5.45) |  | 2.27 | (2.01, 2.55) |  | 2.58 | (2.26, 2.95) |
|  | Q4 | 3.80 | (2.64, 5.47) |  | 5.34 | (3.76, 7.58) |  | 3.39 | (3.04, 3.78) |  | 3.35 | (2.94, 3.81) |
|  | Q5 | 11.21 | (7.71, 16.29) |  | 8.45 | (5.82, 12.26) |  | 5.54 | (4.98, 6.17) |  | 7.49 | (6.60, 8.50) |
| Urban Birth | Q1 | 1.32 | (0.92, 1.88) |  | 3.41 | (1.66, 6.99) |  | 1.49 | (1.34, 1.66) |  | 1.14 | (0.86, 1.51) |
|  | Q2 | 2.45 | (1.80, 3.33) |  | 2.70 | (1.39, 5.25) |  | 1.70 | (1.53, 1.90) |  | 1.43 | (1.13, 1.82) |
|  | Q3 | 3.35 | (2.47, 4.53) |  | 3.25 | (1.81, 5.80) |  | 2.40 | (2.17, 2.65) |  | 2.06 | (1.67, 2.54) |
|  | Q4 | 4.02 | (3.00, 5.39) |  | 6.32 | (3.60, 11.10) |  | 3.27 | (2.96, 3.60) |  | 3.28 | (2.74, 3.93) |
|  | Q5 | 11.18 | (8.13, 15.37) |  | 9.87 | (5.60, 17.37) |  | 6.20 | (5.63, 6.82) |  | 5.80 | (4.87, 6.92) |
| Urban Residence | Q1 | 1.26 | (0.90, 1.78) |  | 2.56 | (1.27, 5.15) |  | 1.50 | (1.35, 1.66) |  | 0.98 | (0.72, 1.33) |
|  | Q2 | 2.37 | (1.77, 3.18) |  | 2.66 | (1.42, 4.98) |  | 1.71 | (1.55, 1.90) |  | 1.24 | (0.96, 1.61) |
|  | Q3 | 3.14 | (2.36, 4.16) |  | 3.62 | (1.92, 6.81) |  | 2.41 | (2.19, 2.65) |  | 1.74 | (1.39, 2.17) |
|  | Q4 | 4.34 | (3.27, 5.76) |  | 5.77 | (3.41, 9.77) |  | 3.34 | (3.04, 3.67) |  | 2.76 | (2.28, 3.34) |
|  | Q5 | 9.64 | (7.24, 12.85) |  | 10.19 | (5.59, 18.58) |  | 6.30 | (5.75, 6.90) |  | 4.99 | (4.15, 6.00) |

IRR, Incidence rate ratio; CI ,Confidence Interval. Model estimates corresponding to figure S4. The reference is no affected relatives and no exposure.

# Supplementary Figures

Figure S1 Mean proportions of type of affected relative by FGRS quintile by case-control status. Lines indicate the type of affected relative including parents (50% genetic similarity), full siblings (50% genetic similarity), 25% genetic similarity (i.e., grandparents, aunt, uncle etc), and those with <=12.5% genetic similarity (cousins, great-grandparents etc).

|  | **FGRS** | **Perinatal factor** | **Prenatal infection** | **Childhood infection** | **ACEs** | **Substance use** | **Urban birth** | **Urban reisdence** |
| --- | --- | --- | --- | --- | --- | --- | --- | --- |
|  | **SCZ** | | | | | | | |
| **FGRS** |  | 0,014 | 0,005 | 0,013 | 0,073* | 0,036* | 0,004 | 0,002 |
| **Perinatal factor** | 0,004 |  | 0,063* | 0,036* | 0,02* | 0,008 | -0,004 | -0,006 |
| **Prenatal infection** | 0,005 | 0,055* |  | 0,054* | -0,006 | -0,006 | -0,007 | -0,011 |
| **Childhood infection** | 0,015* | 0,027* | 0,043* |  | 0,056* | 0,015 | 0,03* | 0,034* |
| **ACEs** | 0,068* | 0,024* | 0,007 | 0,045* |  | 0,135 | 0,057* | 0,049* |
| **Substance use** | 0,042* | 0,011* | 0,004 | 0,026* | 0,111* |  | 0,039* | 0,032* |
| **Urban birth** | 0,009* | -0,001 | 0,008 | 0,014 | 0,035* | 0,018* |  | 0,789* |
| **Urban reisdence** | -0,001 | -0,002 | 0,002 | 0,014 | 0,028* | 0,018* | 0,801* |  |
|  | **BD** | | | | | | | |

Figure S2 Correlations between FGRS and exposure variables .

*p<0.01

Figure S3 IRR for SCZ and BD for exposures unadjusted and adjusted for FGRS quintiles.
Estimates for exposures are relative to no exposure. Points represent IRR, and bars show the corresponding 95% confidence intervals.

Figure S4 IRR for SCZ and BD for exposures unadjusted and adjusted for Parental SCZ/BD (sensitivity analysis).
Estimates for exposures are relative to no exposure. Points represent IRR, and bars show the corresponding 95% confidence intervals.

Figure S5 Interaction models (sensitivity analysis). IRR FOR SCZ (left) AND BD (right) relative to no affected relatives and no exposure for each FGRS QUINTILe. the IRR estimates for exposed (solid line) versus non-exposed (dashed line) for each FGRS quintile. LRT P-values for the adjusted versus interaction models. solid lines represent IRR, and the shaded areas show the corresponding 95% confidence intervals. Axes are on the log-scale.

**References**

1.	Lewis, S.W. and R.M. Murray, *Obstetric complications, neurodevelopmental deviance, and risk of schizophrenia.* Journal of psychiatric research, 1987. **21**(4): p. 413-421.

2.	Scott, J., et al., *Exposure to obstetric complications and subsequent development of bipolar disorder: systematic review.* The British Journal of Psychiatry, 2006. **189**(1): p. 3-11.

3.	Cannon, M., P.B. Jones, and R.M. Murray, *Obstetric complications and schizophrenia: historical and meta-analytic review.* American Journal of Psychiatry, 2002. **159**(7): p. 1080-1092.

4.	SCB. *Folkmängden i Sveriges kommuner 1950-2021*. 2022; Available from: <https://www.scb.se/hitta-statistik/statistik-efter-amne/befolkning/befolkningens-sammansattning/befolkningsstatistik/pong/tabell-och-diagram/helarsstatistik--kommun-lan-och-riket/folkmangden-i-sveriges-kommuner-19502021-enligt-indelning-1-januari-2022/>.

5.	Ludvigsson, J.F., et al., *The longitudinal integrated database for health insurance and labour market studies (LISA) and its use in medical research.* Eur J Epidemiol, 2019. **34**(4): p. 423-437.
